# Association between tea drinking and disability levels in older Chinese adults: a longitudinal analysis

**DOI:** 10.3389/fnut.2023.1233664

**Published:** 2023-10-31

**Authors:** Yinghui Ma, Yuying Zhu, Dandan Hong, Haiyue Zhao, Lei Li

**Affiliations:** ^1^School of Economics and Management, Jiangsu University of Science and Technology, Zhenjiang, China; ^2^School of Economics and Management, Beijing Forestry University, Beijing, China; ^3^College of Economics and Management, Zhejiang A & F University, Hangzhou, China; ^4^Research Academy for Rural Revitalization of Zhejiang, Zhejiang A & F University, Hangzhou, China

**Keywords:** tea drinking, disability levels, elderly, IADL, BADL

## Abstract

**Objective:**

As the global population ages, disability among the elderly presents unprecedented challenges for healthcare systems. However, limited research has examined whether dietary interventions like tea consumption may alleviate and prevent disability in older adults. As an important dietary therapy, the health benefits of tea drinking have gained recognition across research disciplines. Therefore, this study aimed to investigate the association between tea drinking habits and disability levels in the elderly Chinese population.

**Methods:**

Leveraging data from the 2008 to 2018 waves of the Chinese Longitudinal Healthy Longevity Survey, we disaggregated tea drinking frequency and activities of daily living (ADL) measures and deployed fixed-effect ordered logit models to examine the tea-disability association for the first time. We statistically adjusted for potential confounders and conducted stratified analyses to assess heterogeneity across subpopulations.

**Results:**

Multivariable fixed-effect ordered logistic regression suggested tea drinking has protective effects against ADL disability. However, only daily tea drinking was associated with lower risks of basic activities of daily living (BADL) disability [odds ratio (OR) = 0.61; 95% confidence interval (CI), 0.41–0.92] and lower levels of instrumental activities of daily living (IADL) disability (OR = 0.78; 95% CI, 0.64–0.95). Stratified analyses indicated heterogeneous effects across age and income groups. Daily tea drinking protected against BADL (OR = 0.26 and OR = 0.28) and IADL disability (OR = 0.48 and OR = 0.45) for adults over 83 years old and high-income households, respectively.

**Conclusion:**

We found that drinking tea almost daily was protective against disability in elderly people, warranting further research into optimal dosages. Future studies should utilize more rigorous causal inference methods and control for confounders.

## 1. Introduction

The growing prevalence of disability among the elderly has become a critical global issue. An estimated 100 million older adults suffer from varying degrees of disability (1). Disability in the elderly refers to the loss or limitation of their ability to perform daily activities and maintain living skills (2). Treatable pathological factors, such as diabetes or malnutrition, may cause these disabilities (3–5). Therefore, interventions such as treating diseases or improving nutrition may enhance disability status in the elderly. Neurodegenerative diseases, Alzheimer’s disease, and other cognitive disorders lead to a decline in memory, thinking, and behavior in the elderly, impacting their daily living skills and independence (6, 7). There is no complete solution for the disability caused by these degenerative diseases in the elderly. Therefore, adopting preventive strategies is crucial.

China faces a severe aging crisis, with disability rates among older adults increasing from 7% in 2015 to 7.45% in 2020 (8, 9). Projections from China’s Old Age Association show the disabled elderly population over 65 will grow from approximately 187 million in 2020 to over 520 million by 2050. The proportion of disabled elderly people in the total population will also continue to rise, with the disabled elderly population accounting for approximately 13.68% of the total elderly population in 2050 (10).

Tea contains antioxidants and bioactive compounds, such as flavonoids, catechins, polyphenols, and gallic acid (11). These substances all have biological activities and can reduce the risk of dementia by alleviating oxidative stress and inhibiting inflammatory responses (12). Using data from adults aged 65 years and above in Taiwan, Chiu et al. (13) found that higher tea intake was associated with lower disability levels in both men and women. Tao et al. (14) demonstrated that frequent iced tea and tea drinking had protective effects against ADL disability in the elderly. In a cross-sectional study in China, a lack of tea consumption was identified as one of the predictors of functional disability by Zhang et al. (15).

Apart from the above studies, little research has explored the health impacts of tea drinking on disability. For over 4,000 years, people in China, widely recognized as the origin of tea cultivation, have noted tea’s health benefits and therapeutic properties. Chinese people have been the world’s largest consumers of tea since 2006 (16). Therefore, this study aimed to determine the association between tea drinking and disability levels in older adults, which is important for reducing disability risks, improving quality of life, and alleviating caregiving burdens on families and society for the rapidly aging population.

## 2. Materials and methods

### 2.1. Study sample

The Chinese Longitudinal Healthy Longevity Survey (CLHLS), initiated in 1998 by the Center for Healthy Aging and Development Studies at Peking University’s National School of Development, aims to examine the health determinants of individuals aged 65 and above. From 1998 to 2018, approximately half of the counties in 23 provinces, cities, and autonomous regions nationwide were randomly chosen for eight successive surveys, culminating in approximately 113,000 household visits. The survey specifics have been elaborated in previous studies (17, 18). The gathered data encompass demographic and residential information, marital status, lifestyle, socioeconomic attributes, health status, and an extensive array of personal data pertaining to the elderly.

To compensate for participant attrition due to death or loss to follow-up, the CLHLS incorporated new participants, mirroring the characteristics of the departed, to maintain study consistency. All surveys were administered through in-person interviews at the participants’ residences, with each participant providing a signed informed consent form. In cases in which a participant was incapable of signing, a close relative signed on their behalf. This study adhered to the principles of the Declaration of Helsinki.

**FIGURE 1:**
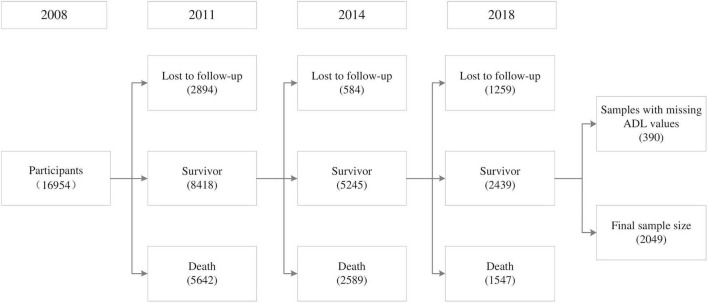
Participant inclusion and exclusion criteria.

Data for this study were gleaned from four CLHLS surveys conducted in 2008, 2011, 2014, and 2018, which included the participants who were surveyed about their tea consumption at baseline and followed up afterward. The sample selection process is shown in Figure 1.

### 2.2. Dependent variables

This study used the Katz Index to gauge disability levels among elderly participants, incorporating both basic activities of daily living (BADL) and instrumental activities of daily living (IADL). BADL ability was evaluated based on six items: bathing, dressing, transferring, toileting, eating, and continence. If the respondent could complete an activity without assistance, it was coded as 1 and otherwise as 0, with scores ranging from 0 to 6. A new variable, “BADL,” was calculated as the sum, in which a maximum score was assigned “1” (representing “non-disabled”); a score of 4–5 was assigned “2” (representing “mild disability”); a score of 2–3 was assigned “3” (representing “moderate disability”); and a score of 0–1 was assigned “4” (representing “severe disability”).

The IADL assessment included eight items: visiting neighbors, shopping, cooking, washing, walking 1 km continuously, lifting 5 kg, crouching and standing up three times continuously, and taking public transport alone. Each item was coded as 1 if the respondent could complete it independently and 0 otherwise, with scores ranging from 0 to 8. A new variable “IADL” was calculated as the sum, in which a maximum score was assigned “1” (representing “non-disabled”); a score of 6–7 was assigned “2” (representing “mild disability”); a score of 3–5 was assigned “3” (representing “moderate disability”); and a score of 0–2 was assigned “4” (representing “severe disability”) (19–21).

### 2.3. Independent variable

The drinking of tea was measured by the question: “How often do you drink tea?” The response options include almost daily; not every day but at least once a week; not every week but at least once a month; not every month but sometimes; and rarely or never. As the study’s independent variable, drinking tea almost daily was assigned a value of 5; not every day but at least once a week was assigned 4; not every week but at least once a month was assigned 3; not every month but sometimes was assigned 2; and rarely or never was assigned 1. Higher scores indicate a higher frequency of tea drinking.

### 2.4. Covariate measurements

This study critically examines several potential covariates, including sociodemographic factors, socioeconomic status, and health-related behaviors. Sociodemographic characteristics mainly included age (65–75, 75–83, and 83+), gender (male or female), marital status (married or unmarried), education level (illiteracy, primary school or below, or junior high school or above), and residential category (urban, town, or rural). For socioeconomic status, whether retired with a pension (yes or no), financial support (dependence or independence) and household income (≤ RMB 20,000 or > RMB 20,000) were mainly considered. For health behaviors, self-rated health (good, normal, and bad), current alcohol drinking and smoking (yes or no), fruit and vegetable consumption (often, occasionally, or rarely), and physical exercise (yes or no) were mainly considered.

### 2.5. Statistical analysis

In the 2008 survey, 16,954 respondents participated and were interviewed. In subsequent follow-ups, 4,737 individuals were lost to follow-up, 9,778 died, and 390 had missing ADL scores; these samples were excluded. A total of 2,049 participants completed the survey four times and were eventually included in the balanced panel data analysis (Figure 1). There were 623 missing values for sociodemographic characteristics and socioeconomic status, which we replaced with the individual’s data from the previous year.

Based on the baseline characteristics summarized by tea consumption frequency, we employed the chi-square test and univariate analysis to examine categorical variables, and variance analysis for continuous variables. Taking into account time-invariant individual characteristics, we utilized panel data methods and applied the fixed effect ordered logit model to assess the odds ratio (OR) and 95% confidence interval (95% CI) for the impact of tea consumption on the level of disability in the elderly during the 2008–2018 follow-up period. Model 1 was a univariate analysis without adjustment. Model 2 adjusted for age, gender, marital status, education level, and residence. Model 3 added retirement status, financial support, self-rated health, drinking, smoking, exercise, and fruit and vegetable intake. For subgroup analyses, we grouped age and income by terciles and dichotomy, respectively, and all models adjusted for demographics, socioeconomics, and health behaviors.

Analyses were performed in STATA 17.0. All *p*-values are two-sided with *p* < 0.05 considered statistically significant.

## 3. Results

### 3.1. Descriptive characteristics

Table 1 displays the baseline descriptive characteristics of the study cohort categorized by tea consumption frequency. The sample consisted of 2,049 respondents with an average age of 75.07 years; 1,072 were female (52.32%), 61.01% were married, and 12.49% resided in urban areas, with the rest living in rural areas. Although the vast majority of the elderly were financially independent (78.28%), 74.82% of them belonged to households with an annual income of less than RMB 20,000. The proportion of the elderly who perceived their health status as good was 56.91% and the proportions of the elderly who smoked and drank were relatively low at 22.99 and 22.69%, respectively. Almost everyone (91.61%) frequently consumed fresh vegetables; only 42.41% ate fresh fruits often and 34.46% engaged in physical exercise. In the total sample, 48.32% of the participants drank tea almost every day and 36.07% rarely or never drank tea. The remaining three categories accounted for only 15.62% of the total sample.

**TABLE 1:** Baseline characteristics of participants stratified by tea drinking frequency.

		Tea frequency					
Characteristics	*N* (%)	Drink little or never	Sometimes drink	Drink at least once a month	Drink at least once a week	Almost every day	*P*
**Gender**							0.000
Male	977 (47.68)	437 (44.73)	56 (5.73)	20 (2.05)	63 (6.45)	401 (41.04)	
Female	1,072 (52.32)	302 (28.17)	59 (5.50)	32 (2.99)	90 (8.40)	589 (54.94)	
**Age group**							0.127
65–75	1,213 (59.20)	446 (36.77)	64 (5.28)	31 (2.56)	99 (8.16)	573 (47.24)	
76–83	526 (25.67)	183 (34.79)	39 (7.41)	15 (2.85)	30 (5.70)	259 (49.24)	
83+	310 (15.13)	110 (35.48)	12 (3.87)	6 (1.94)	24 (7.74)	158 (50.97)	
**Marital status**							0.000
Married	1,250 (61.01)	505 (40.40)	74 (5.92)	27 (2.16)	94 (7.52)	559 (44.72)	
Unmarried	790 (38.56)	234 (29.62)	41 (5.19)	25 (3.16)	59 (7.47)	431 (54.56)	
**Residence**							0.000
Urban	256 (12.49)	96 (37.50)	24 (9.38)	2 (0.78)	16 (6.25)	118 (46.09)	
Town	414 (20.20)	207 (50.00)	17 (4.11)	10 (2.42)	37 (8.94)	143 (34.54)	
Rural	1,379 (67.30)	436 (31.62)	74 (5.37)	40 (2.90)	100 (7.25)	729 (52.86)	
**Education**							0.000
Illiteracy	962 (46.95)	292 (30.35)	48 (4.99)	28 (2.91)	73 (7.59)	521 (54.16)	
Primary school or below	786 (38.36)	307 (39.06)	47 (5.98)	18 (2.29)	60 (7.63)	354 (45.04)	
Junior high school or above	301 (14.69)	140 (46.51)	20 (6.64)	6 (1.99)	20 (6.64)	115 (38.21)	
**Financial**							0.000
Independence	1,604 (78.28)	614 (38.28)	95 (5.92)	40 (2.49)	118 (7.36)	737 (45.95)	
Dependence	445 (21.72)	125 (28.09)	20 (4.49)	12 (2.70)	35 (7.87)	253 (56.85)	
**Income (RMB)**							0.105
≤20,000	1,533 (74.82)	524 (34.18)	86 (5.61)	40 (2.61)	109 (7.11)	774 (50.49)	
>20,000	516 (25.18)	215 (41.67)	29 (5.62)	12 (2.33)	44 (8.53)	216 (41.86)	
**Exercise**							0.004
Yes	706 (34.46)	279 (39.52)	42 (5.95)	25 (3.54)	49 (6.94)	311 (44.05)	
No	1,343 (65.54)	460 (34.25)	73 (5.44)	27 (2.01)	104 (7.74)	679 (50.56)	
**Drinking**							0.000
Yes	465 (22.69)	230 (49.46)	23 (4.95)	10 (2.15)	31 (6.67)	171 (36.77)	
No	1,584 (77.31)	509 (32.13)	92 (5.81)	42 (2.65)	122 (7.70)	819 (51.70)	
**Smoking**							0.000
Yes	471 (22.99)	214 (45.44)	25 (5.31)	7 (1.49)	35 (7.43)	190 (40.34)	
No	1,578 (77.01)	525 (33.27)	90 (5.70)	45 (2.85)	118 (7.48)	800 (50.70)	
**Health**							0.000
Good	1,166 (56.91)	466 (39.97)	62 (5.32)	36 (3.09)	84 (7.20)	518 (44.43)	
Normal	61 (29.33)	202 (33.61)	37 (6.16)	10 (1.66)	50 (8.32)	302 (50.25)	
Bad	282 (13.76)	71 (25.18)	16 (5.67)	6 (2.13)	19 (6.74)	170 (60.28)	
**Work**							0.000
Yes	391 (19.08)	180 (46.04)	39 (9.97)	6 (1.53)	27 (6.91)	139 (35.55)	
No	1,658 (80.92)	559 (33.72)	76 (4.58)	46 (2.77)	126 (7.60)	851 (51.33)	
**Fruit**							0.000
Often	869 (42.41)	361 (41.54)	49 (5.64)	18 (2.07)	56 (6.44)	385 (44.30)	
Occasionally	781 (38.12)	258 (33.03)	69 (8.83)	29 (3.71)	47 (6.02)	258 (33.03)	
Rarely	399 (19.47)	120 (30.08)	19 (4.76)	5 (1.25)	28 (7.02)	227 (56.89)	
**Vegetable**							0.000
Often	1,877 (91.61)	682 (36.33)	105 (5.59)	44 (2.34)	133 (7.09)	913 (48.64)	
Occasionally	150 (7.32)	50 (33.33)	8 (5.33)	8 (5.33)	19 (12.67)	65 (43.33)	
Rarely	22 (1.07)	7 (31.82)	2 (9.09)	0 (0.00)	1 (4.55)	12 (54.55)	
**BADL**							0.000
Non-disabled	2,002 (97.71)	726 (36.26)	111 (5.54)	50 (2.50)	148 (7.39)	967 (48.30)	
Mild disability	36 (1.76)	8 (22.22)	3 (8.33)	2 (5.56)	3 (8.33)	20 (55.56)	
Moderate disability	10 (0.49)	4 (40.00)	1 (10.00)	0 (0.00)	2 (20.00)	3 (30.00)	
Severe disability	1 (0.05)	1 (100.00)	0 (0.00)	0 (0.00)	0 (0.00)	0 (0.00)	
**IADL**							0.000
Non-disabled	1,431 (69.84)	539 (37.67)	85 (5.94)	38 (2.66)	105 (7.34)	664 (46.40)	
Mild disability	329 (16.06)	122 (37.08)	17 (5.17)	6 (1.82)	26 (7.90)	158 (48.02)	
Moderate disability	164 (8.00)	41 (25.00)	7 (4.27)	6 (3.66)	13 (7.93)	97 (59.15)	
Severe disability	125 (6.10)	37 (29.60)	6 (4.80)	2 (1.60)	9 (7.20)	71 (56.80)	
Total (*N*)	2,049 (100.00)	739 (36.07)	115 (5.61)	52 (2.54)	153 (7.47)	990 (48.32)	

Education: education level, divided into illiterate, primary school and below, and junior high school and above. Financial: whether elderly peoples’ sources of income are sufficient, using dependence and independence to indicate whether elderly people are economically independent. Income: the annual income of the whole family, grouped by median. Work: whether they enjoy pension or retirement benefits. Health: self-rated health status. IADL, instrumental activities of daily living; BADL, basic activities of daily living. Yes and No indicate whether a certain activity was performed.

Those who did not benefit from a retirement system were more likely to drink tea almost daily than those who did, a difference that is statistically significant (*p* = 0.000). Similarly, individuals with higher educational levels and those who did not smoke, drink, and regularly consume fresh fruits and partake in other healthy behaviors were more likely to drink tea, with these differences also being statistically significant (*p* < 0.01). Only 2.29% of all participants suffered from varying degrees of BADL disability, whereas the incidence rate of IADL disability was as high as 30.16%. Those without disabilities were more inclined to consume tea.

### 3.2. Logistic regression results

In Table 2, unadjusted fixed effects ordered logit models indicated almost everyday tea drinking protected against BADL (OR = 0.53; 95% CI, 0.34–0.83) and IADL disability (OR = 0.74; 95% CI, 0.61–0.89) compared with drink little or never. After adjusting for sociodemographic characteristics, socioeconomic status, and healthy behaviors, almost everyday tea drinking still had a protective effect against IADL (OR = 0.78; 95% CI, 0.64–0.95) and BADL disability (OR = 0.61; 95% CI, 0.41–0.92).

**TABLE 2:** Associations between tea drinking and disability among older Chinese people.

	IADL (OR 95% CI)		
Item (*n*)	Model 1	Model 2	Model 3
**Tea frequency [drink little or never (2,175)]**			
Almost every day (4,869)	0.74 (0.61, 0.89)***	0.74 (0.60, 0.91)***	0.78 (0.64, 0.95)**
Drink at least once a week (451)	1.12 (0.88, 1.43)	1.13 (0.85, 1.49)	1.12 (0.87, 1.44)
Drink at least once a month (193)	0.91 (0.56, 1.49)	0.89 (0.58, 1.37)	0.95 (0.60, 1.54)
Sometimes drink (508)	0.99 (0.76, 1.30)	0.99 (0.74, 1.33)	1.00 (0.76, 1.34)
	**BADL (OR 95% CI)**		
**Item (*n*)**	**Model 1**	**Model 2**	**Model 3**
**Tea frequency [drink little or never (2,175)]**			
Almost every day (4,869)	0.53 (0.34, 0.83)***	0.50 (0.32, 0.77)***	0.61 (0.41, 0.92)***
Drink at least once a week (451)	1.19 (0.67, 2.11)	1.20 (0.71, 2.05)	1.05 (0.66, 1.69)
Drink at least once a month (193)	0.58 (0.23, 1.45)	0.61 (0.25, 1.47)	0.72 (0.30, 1.71)
Sometimes drink (508)	1.26 (0.71, 2.24)	1.26 (0.70, 2.27)	1.35 (0.75, 2.42)

*** and ** represent 1 and 5% significance levels, respectively. OR, odds ratio; 95% CI, 95% confidence interval. All three models controlled for fixed effects of year and province. The first model did not adjust for any covariates. The second model controlled for demographic and socioeconomic characteristics. The third model built upon the second model by further adding health behaviors and other related variables. *n* is the number of individuals grouped after the total sample size over 4 years.

### 3.3. Subgroup analysis

Previous studies indicate that tea consumption effects on cognition vary by age and economic status in the elderly (22, 23). This section explores whether tea-disability associations differ by age and income. Stratified regression by age (Table 3) showed that drinking frequencies above drink at least once a month have a protective effect against BADL disability in elderly individuals aged 83 and above (OR = 0.36, OR = 0.42, and OR = 0.26). At least once a month (OR = 0.51; 95% CI, 0.2–0.91) and almost everyday drinking (OR = 0.48; 95% CI, 0.33–0.69) were also associated with less IADL disability. These findings indicate that tea drinking may help prevent ADL ability decline and functional deterioration in the oldest section of the population.

**TABLE 3:** The association between tea drinking and disability stratified by age.

	IADL (OR 95% CI)		
Item (*n*)	65–75 (2,948)	76–83 (2,766)	83+ (2,482)
**Tea-frequency (drink little or never)**			
Almost every day	1.16 (0.75, 1.77)	0.67 (0.45, 1.00)*	0.48 (0.33, 0.69)***
Drink at least once a week	1.29 (0.71, 2.35)	0.95 (0.47, 1.89)	0.89 (0.66, 1.21)
Drink at least once a month	1.21 (0.45, 3.30)	1.30 (0.47, 3.60)	0.51 (0.28, 0.91)**
Sometimes drink	1.35 (0.77, 2.38)	0.47 (0.21, 1.04)*	0.73 (0.42, 1.24)
	**BADL (OR 95% CI)**		
**Item (*n*)**	**65–75 (2,948)**	**76–83 (2,766)**	**83+ (2,482)**
**Tea frequency (drink little or never)**			
Almost every day	2.12 (0.65, 6.92)	0.42 (0.16, 1.07)*	0.26 (0.16, 0.44)***
Drink at least once a week	2.55 (0.65, 9.95)	3.14 (0.72, 13.67)	0.42 (0.29, 0.62)***
Drink at least once a month	0.51 (0.07, 3.93)	0.31 (0.02, 4.23)	0.36 (0.13, 0.98)**
Sometimes drink	11.12 (1.24, 99.40)	0.64 (0.04, 9.51)	0.55 (0.26, 1.16)

***, **, and * represent 1, 5, and 10% significance levels, respectively. OR, odds ratio; 95% CI, 95% confidence interval. The models all controlled for confounding factors such as demographic characteristics, socioeconomic status, health behaviors, and other related variables. All the models controlled for fixed effects of year and province. *n* is the number of individuals grouped after the total sample size over 4 years.

Income was dichotomized into high and low groups. Stratified analysis (Table 4) showed that almost everyday tea drinking had a positive protective effect against BADL disability in high-income elderly people (OR = 0.28; 95% CI, 0.15–0.50). Sometimes and almost everyday drinking also protected against IADL disability decline (OR = 0.78 and OR = 0.45, respectively). However, tea drinking did not significantly impact BADL or IADL disability in low-income elderly people.

**TABLE 4:** The association between tea drinking and disability stratified by income.

	IADL (OR 95% CI)	
Item (*n*)	Low income (4,600)	High income (3,596)
**Tea frequency (drink little or never)**		
Almost every day	0.89 (0.66, 1.19)	0.45 (0.29, 0.70)***
Drink at least once a week	0.91 (0.58, 1.43)	0.80 (0.50, 1.27)
Drink at least once a month	1.17 (0.63, 2.18)	0.86 (0.45, 1.66)
Sometimes drink	1.11 (0.73, 1.69)	0.78 (0.35, 1.76)***
	**BADL (OR 95% CI)**	
**Item (*n*)**	**Low income (4,600)**	**High income (3,596)**
**Tea frequency (drink little or never)**		
Almost every day	0.83 (0.34, 2.04)	0.28 (0.15, 0.50)***
Drink at least once a week	1.14 (0.36, 3.55)	0.94 (0.50, 1.79)
Drink at least once a month	1.54 (0.36, 6.68)	0.30 (0.61, 1.52)
Sometimes drink	2.07 (0.83, 5.15)	0.64 (0.21, 1.98)

*** represents a 1% significance level. OR, odds ratio; 95% CI, 95% confidence interval. The models all controlled for confounding factors such as demographic characteristics, socioeconomic status, health behaviors, and other related variables. All the models controlled for fixed effects of year and province. *n* is the number of individuals grouped after the total sample size over 4 years.

## 4. Discussion

In this large prospective elderly cohort, we found that frequent tea drinking (almost daily) could alleviate the deterioration of daily living activities and lower risks of BADL and IADL disability, whereas occasional tea drinking did not provide protective effects. Our findings provide new evidence that frequent tea consumption could help prevent disability in the elderly.

In the context of widespread tea culture, tea drinking has always been considered as a promising non-pharmacological health strategy and has always been used to assist the management of hypertension, obesity, and diabetes (24). Tea and its components have been extensively used in functional foods and supplements to prevent and treat diverse conditions, including Parkinson’s disease (25), stroke (26), and dementia (12), among others. Certain tea types, including black tea and oolong tea, contain abundant tea polyphenols like catechins and theaflavins, conferring beneficial properties such as antioxidant, anti-inflammatory, and neuroprotective activities that positively associate with health-related quality of life in aging populations (27). White tea is abundant in xanthine glycosides, flavonoid glycosides, and methylated flavan-3-ols, exhibiting antihypertensive and antioxidant actions (28). Rodent experiments by Li et al. (29) revealed white tea extract (WTE) can modulate lipid metabolism in cultured rat adipocytes and hepatocellular carcinoma cells. Shen et al. (30) reported green tea epigallocatechin and epicatechin can stimulate bone formation by modulating osteoblast differentiation and proliferation. These catechins also suppress inflammation and bone resorption to benefit bone health. Animal studies have demonstrated various protective effects of green tea, including antioxidant, anti-inflammatory, antihypertensive, antidiabetic, and hepatoprotective activities (31–33). Sustained tea intake may positively impact physiological health (34), thereby improving quality of life (35) and promoting healthy aging. Consequently, tea drinking may play an important role in disability protection for the elderly population.

The findings of this study demonstrated protective effects of tea drinking on BADL and IADL disability and that almost daily tea drinking was associated with lower levels of disability in older people, a finding that contrasts with several previous studies. Tea contains a variety of molecular compounds, including caffeine, oxalic acid, and tannic acid. Numerous scientific studies have shown that excessive caffeine intake can lead to insomnia, anxiety, rapid heartbeat, and other harmful effects (36). Additionally, high consumption of oxalic acid may adversely impact kidney function (37). The tannic acid in tea may be associated with reduced iron absorption (38). Additionally, lack of sleep and poor kidney function can increase the risk of disability in the elderly (39, 40).

Contrary to the potential negative effects noted earlier, some research indicates that adequate and regular tea drinking may promote health benefits in the elderly population (41). Elderly individuals who regularly drink tea have better cognitive function, and regular tea drinking (at least once a week) is associated with a lower risk of cognitive impairment (22, 42). A diminished capacity for activities of daily living among older adults is associated with cognitive decline (43), which critically predicts functional impairment and disability in this population (44, 45). Elderly people with greater cognitive function encounter a lower disability risk (46), suggesting that tea drinking may reduce disability prevalence by protecting against cognitive decline.

Cognitive decline in elderly people is mainly caused by three factors: neurodegenerative diseases (47), neurotransmitter imbalances (48), and brain atrophy (49), which often have complex interactions with oxidative stress and inflammation. Tea possesses abundant bioactive constituents associated with anti-inflammatory and antioxidant mechanisms (13, 50), which are capable of conferring antioxidant, anti-inflammatory, and neuroprotective properties that may enhance cognitive health and mitigate disability risk in older populations. Tea polyphenols manifest antioxidant, anti-inflammatory, and neuroprotective qualities that help reduce oxidative damage, suppress neuroinflammation, and protect neurons from harm (51). Catechins alleviate oxidative stress and inflammation, decelerating the progression of neurodegenerative diseases (52). Caffeine inhibits brain fatty acid amide hydrolase and decreases amyloid-beta accumulation, thus reducing susceptibility to neurodegenerative conditions like Alzheimer’s disease (53). L-theanine has anxiolytic and antidepressant effects that can alleviate cognitive impairment by regulating neurotransmitters such as GABA, serotonin, and dopamine (54), partially validating the foregoing analysis.

Our stratified regression analysis showed that tea drinking was associated with higher BADL and IADL scores in those over 83 years old. This aligns with previous studies showing that frequent tea drinking is associated with better cognition in Chinese octogenarians (22) and improves cognition in Japanese people over 70 years old who consume green tea (55). Advanced age confers a risk for disability (56). Statistics indicate that the disability rate is nearly three times higher in Chinese people aged 75–84 versus 65–74, with nearly half of the over 85 s eventually developing total disability (57). Our findings suggest that tea consumption helps prevent disability in the very old. IADL represents the ability needed for social interaction activities in middle-aged and elderly people, who are more sensitive to subtle functional defects (58). Assessing IADL enables the early detection of physical and cognitive decline in elderly people, allowing timely intervention.

Economic status influences diet (59, 60). Tea consumption and associated health benefits also depend on income (61). Thus, we conducted income-stratified analyses (high vs. low), controlling for socioeconomic factors. The results revealed differing tea-disability patterns by income. Drinking tea almost every day has a protective effect against IADL and BADL disability in elderly people from high-income households, whereas no significant impact was observed in low-income elderly people. This may reflect higher stroke, dementia, and disability rates among those of a lower economic status (62, 63). Although beneficial, tea alone may be insufficient as treatment. Higher tea consumption frequency and quality among high-income elderly likely stems from health motivations, which confer preventive effects (64).

The advantage of our study is the use of a fixed effects ordered logit model to analyze panel data. This controlled for individual heterogeneity and provided more reliable association measurements than Cox models. This model also allowed us to evaluate the treatment effect of tea drinking within individuals, overcoming the limitations of previous studies that did not control for time-invariant unobserved characteristics (65–67). Furthermore, we refined the independent and dependent variables to identify the tea consumption frequency with the greatest impact on disability in the elderly and quantify the protective effects of different frequencies. Using both IADL and BADL provided comprehensive assessments of disability; IADL was more sensitive in capturing mild disability and early functional decline, helping timely discovery and intervention. Our conclusions show that only drinking tea almost every day confers protective effects against disability, rather than casual tea drinking. This study has several limitations. First, owing to the limitations of the questionnaire items, this study used the frequency of drinking tea as the independent variable, and the questionnaire did not include questions regarding the types and doses of tea intake. Future studies can explore the effects of different types of tea and tea doses on disability in elderly people. Second, although this paper selected the ADL scale as an indicator of disability level, the scoring criteria were rather rough due to the limitations of the answer types in the questionnaire.

## 5. Conclusion and recommendations

Tea drinking among older adults was found to help reduce the risk of increased disability levels. Regular tea drinking exerted a certain protective effect against IADL and BADL disability in the elderly population; daily drinking appeared to be the optimal frequency. The protective effects of tea drinking were more pronounced in the very old and high-income elderly. These findings may have important implications for public health efforts aimed at preventing functional capacity decline in older adults. Additional rigorous observational studies are warranted to further elucidate this relationship, accounting for tea type and potential interactive effects.

Disability can profoundly impact the quality of life and mental wellbeing of older adults and impose tremendous socioeconomic burdens (68), as disabled elderly people often require more care, healthcare, and rehabilitation services, undoubtedly increasing the economic burden on families and society (69). Given global aging trends, the number of disabled older adults continues to rise. Long-term care systems are widely deemed to be fundamental for addressing looming crises in the care of elderly people and protecting health in later life. Our findings may also provide new perspectives on developing better long-term care models.

## Data availability statement

Publicly available datasets were analyzed in this study. This data can be found here: https://opendata.pku.edu.cn/dataverse/CHADS.

## Ethics statement

The studies involving human participants were reviewed and approved by the Ethical Review Committee of Peking University. The patients/participants provided their written informed consent to participate in this study.

## Author contributions

YM: obtained funding, conceptualized the study, assisted with statistical analysis, helped conduct the literature search and review, wrote, and revised the manuscript. YZ: conducted the literature search and review, analyzed the data, assisted with statistical analysis, wrote, and revised parts of the manuscript. DH and HZ: conducted statistical analysis, drafted the manuscript, wrote, and revised parts of the manuscript. LL: contributed to the writing of the final version of the manuscript. All authors have read and agreed to the published manuscript.
